# PRL-3 promotes the motility, invasion, and metastasis of LoVo colon cancer cells through PRL-3-integrin β1-ERK1/2 and-MMP2 signaling

**DOI:** 10.1186/1476-4598-8-110

**Published:** 2009-11-24

**Authors:** Lirong Peng, Xiaofang Xing, Weijun Li, Like Qu, Lin Meng, Shenyi Lian, Beihai Jiang, Jian Wu, Chengchao Shou

**Affiliations:** 1 Key Laboratory of Carcinogenesis and Translational Research (Ministry of Education), Department of Biochemistry and Molecular Biology, Peking University School of Oncology, Beijing Cancer Hospital & Institute, Beijing 100142, PR China

## Abstract

**Background:**

Phosphatase of regenerating liver-3 (PRL-3) plays a causative role in tumor metastasis, but the underlying mechanisms are not well understood. In our previous study, we observed that PRL-3 could decrease tyrosine phosphorylation of integrin β1 and enhance activation of ERK1/2 in HEK293 cells. Herein we aim to explore the association of PRL-3 with integrin β1 signaling and its functional implications in motility, invasion, and metastasis of colon cancer cell LoVo.

**Methods:**

Transwell chamber assay and nude mouse model were used to study motility and invasion, and metastsis of LoVo colon cancer cells, respectively. Knockdown of integrin β1 by siRNA or lentivirus were detected with Western blot and RT-PCR. The effect of PRL-3 on integrin β1, ERK1/2, and MMPs that mediate motility, invasion, and metastasis were measured by Western blot, immunofluorencence, co-immunoprecipitation and zymographic assays.

**Results:**

We demonstrated that PRL-3 associated with integrin β1 and its expression was positively correlated with ERK1/2 phosphorylation in colon cancer tissues. Depletion of integrin β1 with siRNA, not only abrogated the activation of ERK1/2 stimulated by PRL-3, but also abolished PRL-3-induced motility and invasion of LoVo cells in vitro. Similarly, inhibition of ERK1/2 phosphorylation with U0126 or MMP activity with GM6001 also impaired PRL-3-induced invasion. In addition, PRL-3 promoted gelatinolytic activity of MMP2, and this stimulation correlated with decreased TIMP2 expression. Moreover, PRL-3-stimulated lung metastasis of LoVo cells in a nude mouse model was inhibited when integrin β1 expression was interfered with shRNA.

**Conclusion:**

Our results suggest that PRL-3's roles in motility, invasion, and metastasis in colon cancer are critically controlled by the integrin β1-ERK1/2-MMP2 signaling.

## Background

Colorectal cancer ranks third in the incidence of cancer in the world, and metastasis is the main death cause. Although causes and genetic bases of tumorigenesis vary greatly, key events required for metastasis are similar, including alteration of adhesion ability, enhancement of motility, and secretion of proteolytic enzymes to degrade extracellular matrix (ECM) and vascular basement membrane; all these steps are orchestrated by a plethora of signaling events. Phosphatase of regenerating liver-3 (PRL-3), also known as PTP4A3, encodes a 22-kilodalton protein tyrosine phosphatase and is characteristic of a CAAX motif for prenylation at the carboxyl terminus [1]. At mRNA level, it is detected primarily in skeletal and cardiac muscles, somewhat in pancreas, but rarely in brain, lungs, liver, kidneys, and placenta [2]. However, it is highly expressed in multiple cancer cell lines and vascular endothelial cells [3-5]. Initially, PRL-3 was found to be up-regulated in liver metastases of colorectal cancer, but was low or absent in normal colorectal epithelium, adenoma, and primary lesions [6]. Later, we and other several groups provided strong evidence to show that PRL-3 is overexpressed in diverse malignancies, including colorectal, breast, gastric, and ovarian cancers, and its expression is correlated with disease progression and survival [7-14]. A recent study by Molleví et al. demonstrated that tumor microenvironment play a critical role in regulating PRL-3 expression[15]. To date, PRL-3 is not only thought as a potential prognostic factor for diagnosis and survival of multiple type cancers, but also has a therapeutic implication, because its expression at the invasive margin of tumor predicted resistance to radiotherapy and unfavorable survival for patients [16,17].

Previous studies also revealed that PRL-3 plays a causative role in promoting cell motility, invasion, and metastasis [18,19]. However, little is known about the molecular mechanisms by which PRL-3 promotes motility, invasion and metastasis. It was reported that PRL-3 exerted its functions by regulating Rho family GTPase [20], activating Src [21], and modulating PI3K-Akt pathway [22] in a context-dependent manner. In addition, a transcriptional regulation of PRL-3 by p53 has been reported [23]. In our previous study, we found a physical association between PRL-3 and integrin α1 by yeast two-hybrid and GST-pull down assays [24]. We also observed decreased tyrosine phosphorylation of integrin β1 and enhanced phosphorylation of extracellular signal-regulated kinase 1/2 (ERK1/2) in exogenous PRL-3-stably expressing HEK293 cells. Integrins is a large family of heterodimeric cell-surface receptors and integrin-mediated extracellular signals stimulate a variety of intracellular signaling events, including tyrosine phosphorylation and mitogen-activated protein kinase (MAPK) cascades, leading to the ERK activation, which is involved in cell survival and proliferation, and promotes metaplasia and tumor development [25-28]. Therefore, in the present study, we investigated the functional roles of integrin signaling and ERK1/2 activation in PRL-3-promoted motility, invasion, and metastasis in colon cancer cell LoVo. We verified the enhancement of ERK1/2 phosphorylation in PRL-3-stably expressing LoVo (LoVo-P) cells. Knockdown of integrin β1 not only inhibited PRL-3-induced ERK1/2 phosphorylation, but also abrogated PRL-3-mediated motility, invasion, and lung metastasis in nude mice. In the downstream of integrin β1 pathway, ERK1/2 phosphorylation and MMP2 activity were found to be responsible for PRL-3-mediated cell invasion. Collectively, our study demonstrated that the integrin β1-ERK1/2 and -MMP2 signaling plays critical roles in PRL-3-promoted motility, invasion, and metastasis of colon cancer cells.

## Methods

### Reagents and cell culture

We purchased anti-integrin β1 anitbody (MAB 1965) from Chemicon (Temecula, CA). Anti-phosphorylated tyrosine antibody 4G10 was from Millipore (Billerica, MA). Monoclonal antibody 3B6 against PRL-3 was generated as previously described [29]. Polyclonal antibody to PRL-3 was from Sigma. Antibodies against ERK1/2 and phosphorylated ERK1/2 (p-ERK1/2) were from Upstate (Beverly, MA). Anti-p53 antibody (DO-1) was from Santa Cruz. U0126 was from Cell Signaling (Beverly, MA). Colon cancer cell line LoVo (ATCC, Manassas, VA) were maintained in Ham's F12K medium (Invitrogen) supplemented with 10% fetal calf serum.

### Plasmids and transfection

Myc-tagged human PRL-3 cDNA was inserted into pcDNA3.1 at BamH I/Xba I sites to generate a mammalian expression plasmid pcDNA3.1-PRL-3. Then, pcDNA3.1-PRL-3 and pcDNA3.1 were transfected into LoVo cells with Lipofectamine 2000 (Invitrogen) to generate PRL-3-stably expressing and control cells, respectively. After 4 weeks of selection with 600 μg/mL of Geneticin (Invitrogen), expression of PRL-3 was verified by RT-PCR and Western blot. Plasmid pEGFP-C1-PRL-3 was generated by ligating BamH I/EcoR I digested full-length PRL-3 to Bgl II/EcoR I digested pEGFP-C1 vector (Clontech, Palo Alto, CA).

### RNA interference

Integrin β1-specific siRNA, synthesized by Sigma-Aldrich Corporation (St. Louis, MO), was designed to silence all splices of human integrin β1 mRNA. The sequence was: sense, 5'-GGAAAUGGUGUUUGCAAGUdTdT-3'; antisense, 5'-ACUUGCAAACACCAUUUCCdTdT-3'. It was scrambled to generate a negative control. Lentivirus vectors expressing short hair-pin (sh)RNA targeting PRL-3 or integrin β1 were constructed, packed, and purified by GeneChem Corporation (Shanghai, China), and was manipulated according to the Biological Institutional Committee of Beijing.

### Western blot and immunoprecipitation

Cells were homogenized in lysis buffer (50 mM Tris-HCl, pH 7.5, 150 mM NaCl, 1% NP-40, 1 mM DTT, 1 mM phenylmethylsulfonyl fluoride, 10 mM NaF, 1 mM Na_3_VO_4_, 1 × protease cocktail) for 20 min at 4°C. The supernatant was collected after centrifugation at 12,000 × g for 20 min at 4°C and subjected to Western blot or immunoprecipitation as previously described [24]. Documentation of blots was performed by scanning with an EPSON PERFECTION 2580 scanner and acquired images were adjusted by the Auto-Contrast command of Photoshop CS (Adobe, San Jose, CA).

### Immunohistochemical analysis

Consecutive 4-μm paraffin-embedded sections of colon cancer tissues were obtained from the Department of Pathology of the Beijing Cancer Hospital and Institute. Staining of PRL-3 or p-ERK1/2 protein by an immunohistochemical assay was performed as previously described [7]. Specimens with more than 10% positive-staining cancer cells were classified as positive.

### Motility and invasion assays

For transwell chamber-based motility and invasion assays, equal amounts of cells were loaded into an insert provided with serum-free medium and allowed to pass through an 8-μm-pore polycarbonate filter, which had been either pre-coated with 100 μg of Matrigel (Becton Dickinson, San Jose, CA) for invasion assay or left uncoated for motility assay. Medium supplemented with 10% fetal calf serum was added to the bottom chamber. Cells on the upper surface of filters were wiped out after 24 h (motility assay) or 48 h (invasion assay), and those on the undersurface were stained with 1% amino toluene blue and counted under a microscope.

### In vitro wound healing assay

Cells were seeded onto 6-well plates at a sub-confluent density. After 12 h, a line was scrapped out on the cell monolayer by a 200-μl pipet tip and the width of this wound line was photographed using an inverted microscope (ECLIPSE TS100, Nikon, Japan) at a 24 h interval. The motility speed of cells was assessed by the healing degree of the wound line. The experiment was repeated three times independently.

### Indirect immunofluorescence

To visualize green fluorescent protein (GFP) tagged PRL-3, LoVo cells were transfected with pEGFP-C1-PRL-3 and seeded onto coverslips. For indirect immunofluorescence assays, pEGFP-C1-PRL-3 transiently transfected LoVo cells were fixed with 4% paraformaldehyde for 10 min at room temperature, permeabilized with 0.5% Triton X-100/phosphate-buffered saline for 5 min, and blocked with 3% bovine serum albumin for 30 min. Anti-integrin β1 antibody was then added to the cells, followed with a tetramethyl rhodamine isothiocyanate-conjugated secondary antibody. After washing with phosphate-buffered saline/Tween-20, coverslips were mounted on glass slides with 50% glycerol/phosphate-buffered saline and imaged using a Leica SP2 confocal system (Leica Microsystems, Dresden, Germany).

### Metastasis study

We performed animal experiments in accordance with the Experimental Animal Management Ordinance approved by the Scientific and Technological Committee of China. Every of the five experimental groups had eight 4 to 6-week-old female nude BALB/c mice (Lian-Tong-Li-Hua Corporation, Beijing, China). Each mouse was injected via tail vein with 2.5 × 10^6 ^LoVo control (LoVo-C) or LoVo-PRL-3 (LoVo-P) cells; the latter were pre-infected with lentivirus interfering with PRL-3, integrin β1, or mock control, respectively. Two months later, all animals were sacrificed, and 4-μM paraffin-embedded sections of lung and liver tissues were prepared. The sections were stained with hematoxylin and eosin and examined for the presence of metastatic tumor foci under a microscope.

### Zymographic analysis

The zymographic analysis was adapted from Surgucheva IG et al. [30]. Cells were grown to 70-80% confluence on 10-cm plates, washed twice with PBS, and cultured in serum-free medium for another 36 h. Next, medium was concentrated to one-tenth volume and measured for protein concentration. Appropriate volume of medium with equivalent amount of protein was subjected to electrophoresis in 10% gel containing 0.1% gelatin. After electrophoresis, the gelatin gel was washed twice with 2.5% Triton X-100 and allowed to perform an enzyme reaction in Tris buffer (50 mM Tris-HCl at pH 7.4, 200 mM NaCl, and 10 mM CaCl_2_) overnight at 37°C. Next, it was stained with 0.5% Coomassie Brilliant Blue R-250 and de-stained with 5% acetic acid containing 10% methanol.

### Statistical analysis

Statistical analysis software package SPSS 12.0 (SPSS Inc., Chicago, IL) was used to perform Poisson distribution events test and Chi-square test. P value less than 0.05 was considered statistically significant.

## Results

### PRL-3 is associated with integrin β1

**Figure 1:**
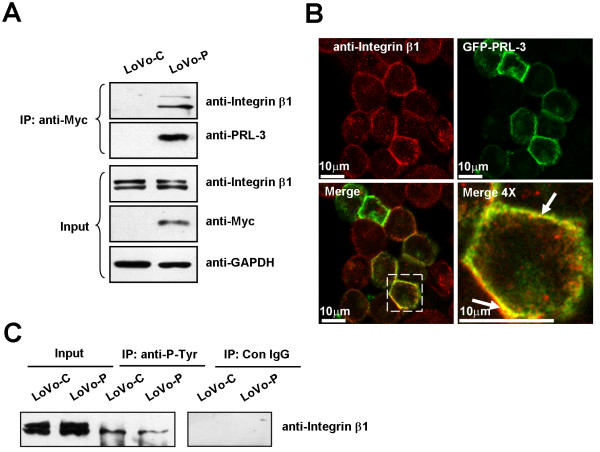
**PRL-3 interacts with integrin β1 and decreases its tyrosine phosphorylation in LoVo cells**. (A) PRL-3 interacted with integrin β1. Equal amount of lysates (500 μg of protein) from LoVo-P cells, which stably expressed myc-tagged human PRL-3, and control LoVo-C cells were immunoprecipitated with anti-myc antibody, followed by Western blot with anti-integrin β1 antibody and anti-PRL-3 antibody. Expression of integrin β1 and PRL-3 in the lysates (50 μg of protein) was shown as Input. GAPDH protein expression was shown as a loading control. Molecular weight was shown. (B) PRL-3 was colocalized with integrin β1. LoVo cells were transiently transfected with GFP-PRL-3 (green). Twenty-four hours after transfection, cells were fixed, stained with an anti-integrin β1 antibody (red), and observed under a laser confocal microscope. The white arrow in Merge (4×) indicates the colocalization of PRL-3 with integrin β1 (yellow). (C) PRL-3 decreased tyrosine phosphorylated integrin β1. Equal amount of lysates (500 μg of preotein) from LoVo-C and LoVo-P cells were immunoprecipitated with anti-phosphotyrosine antibody or IgG control. The precipitates were subjected to Western blot with anti-integrin β1.

In a previous study, we found a physical interaction between PRL-3 and integrin α1 [24]. As a membrane receptor, integrin α1 can heterodimerize with different members of integrin β family, including integrin β1, to initiate signaling transduction. It is usually thought that integrin α subunit is associated with ECM adhesion, while β subunit is mainly responsible for signal transduction [31]. To understand the functional relevance of the association between PRL-3 and integrin signaling, we firstly transfected PRL-3 cDNA or vector control into human colon cancer cell line LoVo, which has no detectable PRL-3 protein expression even in the presence of genotoxic stress by Adriamycin [see Additional file 1], which stabilized p53, a known PRL-3 inducer at transcriptional level [23]. After selection with Geneticin, stable expression of Myc-tagged PRL-3 in LoVo-P cells was verified by Western blot (Figure 1A) and RT-PCR (data not shown). Next, we examined the interaction between PRL-3 and integrin β1 by immunoprecipitating PRL-3 with anti-Myc antibody, followed by immunoblotting with anti-integrin β1. Integrin β1 was observed in precipitates of LoVo-P cells but not in that of LoVo-C cells (Figure 1A), indicating that PRL-3 assocaited with integrin β1 in LoVo cells.

To better support this result, we examined subcellular localization of transiently overexpressed GFP-tagged PRL-3 and endogenous integrin β1 in LoVo cells by an indirect immunofluorescence assay. Figure 1B showed that both GFP-tagged PRL-3 and fluorescent antibody-labeled integrin β1 were expressed in cytoplasmic membrane. Dual-color merged confocal imaging demonstrated that PRL-3 was colocalized with integrin β1 (Figure 1B).

Previously, we noticed a decrease of integrin β1 tyrosine phosphorylation in PRL-3-stably expressing HEK293 cells [24]. Therefore, we checked the effect of PRL-3 on tyrosine phosphorylation of integrin β1 in LoVo cells. Using equal amount of lysates from LoVo-C and LoVo-P cells, we immunoprecipiated tyrosine-phosphorlyated integrin β1 with a phospho-tyrosine specific antibody, respectively, and immunoblotted it with anti-integrin β1 antibody. Tyrosine-phosphorylated integrin β1 was found in both cells, however, it was decreased in LoVo-P cells (Figure 1C), though integrin β1 protein expressed at similar level. This result substantiated PRL-3's role in regulating tyrosine phosphorylation of integrin β1.

### Integrin β1 is necessary for PRL-3-induced cell motility and invasion in vitro

**Figure 2:**
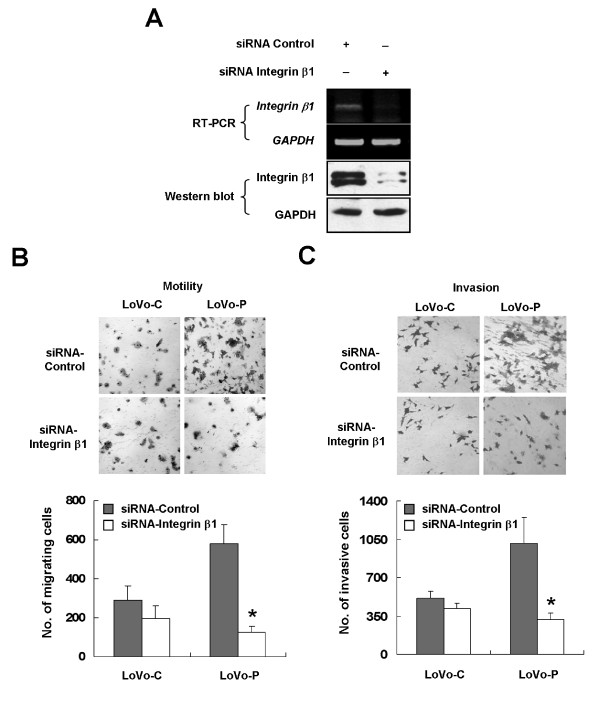
**Integrin β1 mediates PRL-3-induced cell motility and invasion**. (A) LoVo-P cells were treated with siRNA specific for integrin β1 or control. Seventy-two hours after transfection, cells were harvested. Equal amount of protein lysates (50 μg of protein) was analyzed for integrin β1 protein expression and RNA was extracted for RT-PCR. (B) and (C) Integrin β1 is required for PRL-3-induced cell motility and invasion. Cells were transfected with indicated siRNAs as in (A). Seventy-two hours after transfection, cells were analyzed for their motility and invasion with the use of transwell chambers. Cells were suspended in serum-free medium and loaded at a density of 2.5 × 10^4 ^to an insert of a transwell chamber, and those migrating or invading to the underside of filtera were stained and counted after 24 h (B, motility assay) or 48 h (C, invasion assay). Top panel: Representative illustrations for motility and invasion assays (original magnification, × 200). Bottom panel: Quantification of migrating and invasive cells. Values were the total number of stained cells. The experiments were repeated at least three times independently. Error bars represent standard errors of the mean value (*, P < 0.05).

Integrins are involved in diverse malignant phenotypes of tumor, including invasion and metastasis [32]. PRL-3 is also crucial for cancer cell motility, invasion, and metastasis [18]. Considering the association between PRL-3 and integrin β1 found above, we investigated the requirement of integrin β1 for PRL-3-promoted cell motility and invasion. To this end, cells were treated with a small interfering RNA (siRNA) against integrin β1 or a control siRNA. After confirmation of silencing efficiency by RT-PCR and Western blot (Figure 2A), migrating and invasive capacities of LoVo-C and LoVo-P cells were analyzed with transwell chambers as described in Experimental Procedures. Consistent with previous studies with other cancer cell lines [18,19], PRL-3 enhanced LoVo cell motility and invasion (Figure 2B). However, knockdown of integin β1 significantly impaired the migrating and invasive abilities of LoVo-P cells (*, P < 0.05, Figure 2B and 2C), but not those of LoVo-C cells. To further validate these results, we performed wound healing assay. As shown in Additional file 2, the speed of wound healing of LoVo-P cells was faster than that of LoVo-C cells. By 72 h after wounding, the wound of LoVo-P were almost closed up, while those of LoVo-C cells were still wide apart. However integrin β1 inhibition substantially abolished the effect of PRL-3.

### Integrin β1 is required for PRL-3-induced metastasis in vivo

**Figure 3:**
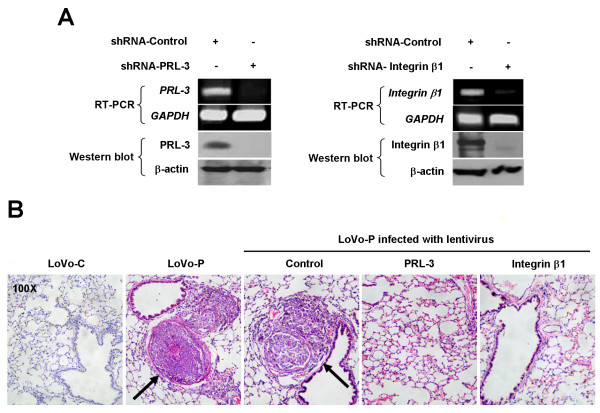
**Depletion of integrin β1 or PRL-3 abrogates PRL-3-promoted metastasis in vivo**. LoVo-P cells were infected with lentivirus interfering with PRL-3, integrin β1, or control respectively at a multiplicity of infection value of 100 for 48 h, or left untreated. (A) Validation of silencing efficiency of Lentivirus by RT-PCR and Western blot. (B) Each nude BALB/c mouse was injected via the tail vein with 2.5 × 10^6 ^LoVo-C or LoVo-P cells. Two months later, mice were sacrificed, and 4-μm paraffin slices of liver and lung tissues were stained with hematoxylin and eosin dyes and examined under a light microscope. Arrows indicate the presence of a metastatic tumor in a lung slice.

Given the role of integrin β1 in mediating PRL-3's in vitro effect on cell motility and invasion, we further examined the requirement of integrin β1 for PRL-3-mediated metastasis in nude mouse BALB/c, which are immunodeficient and susceptible to tumor formation and metastasis. First, lentivirus interfering with expression of PRL-3 or integrin β1 was generated, respectively, and their silencing efficiencies were verified by RT-PCR and Western Blot (Figure 3A). Next, LoVo-P cells were infected with control-, PRL-3-, or integrin β1-interference lentivirus for 48 h. Then each nude mouse was injected via tail vein with 2.5 × 10^6 ^LoVo-C or LoVo-P cells, which had been infected with lentivirus or not as listed in Table 1. Two months later, nude mice were sacrificed and dissected. No macroscopic tumors were found in all organs of the dissected mice (data not shown). Livers and lungs were isolated, fixed with formalin, and prepared for 4-μm paraffin-embedded slices. The slices were stained with hematoxylin and eosin, and subjected to microscopic observation for metastatic foci. No metastatic tumor was found in livers of all groups (data not shown), possibly due to minimally hepatophilic property of LoVo cells. As shown in Table 1, 13 and 8 lung metastatic foci were respectively found in uninfected LoVo-P group and control-lentivirus infected LoVo-P group, whereas none was found in LoVo-C group (*, P < 0.05). Though control-lentivirus infected LoVo-P group formed less metastatic foci than uninfected LoVo-P group, there was no statistical significance (P > 0.05). This difference might result from the side effects of lentivirus infection. However, there was only 1 metastatic tumor formed in PRL-3-interference LoVo-P and integrin β1-interference LoVo-P groups (*, P < 0.05, compared to control-lentivirus LoVo-P group), respectively. The representative illustrations of hematoxylin and eosin staining were demonstrated in Figure 3B. These results support that integrin β1 is crucial for PRL-3-promoted metastasis in vivo.

**Table 1:** Metastasis of LoVo-C and LoVo-P Cells in Nude Mice BALB/c

Group	Total number of mice	Number of mice with metastasis	Number of metastatic foci
LoVo-C uninfected	8	0	0 *
LoVo-P uninfected	8	4	13
LoVo-P lentivirus-control	8	4	8
LoVo-P lentivirus-integrin β1	8	1	1 *
LoVo-P lentivirus-PRL-3	8	1	1 *

*P < 0.05 when compared to LoVo-P lentivirus-control

Statistical analysis was performed with Poisson distribution events test

### Integrin β1 is required for PRL-3-induced ERK1/2 activation

**Figure 4:**
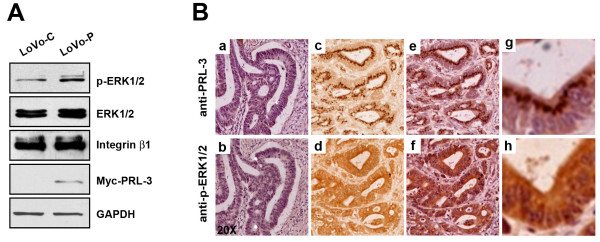
**Correlation between PRL-3 level and ERK phosphorylation**. (A) PRL-3 enhanced ERK1/2 phosphorylation in LoVo cells. Equal numbers of LoVo-C and LovO-P cells were cultured for 24 h with complete medium. Cells were harvested and lyzed. Then cell lysates (50 μg of protein) were subjected to Western blot with antibodies against p-ERK1/2, ERK1/2, Myc-tag (for Myc-PRL-3), integrin β1, and GAPDH, respectively. (B) Expression of PRL-3 and p-ERK1/2 in primary lesions of human colon cancer tissues was analyzed by an immunohistochemical assay. Two consecutive 4-μm paraffin-embedded slices of the same tissue sample were probed with anti-PRL-3 (a, c, e, g) and anti-p-ERK1/2 (b, d, f, h), respectively. The representative negative staining of PRL-3 and p-ERK1/2 in the consecutive slices was shown in a and b, and the representative positive staining of PRL-3 and p-ERK1/2 was shown in the c, d, e, f, g, h (c and d, without HE counterstaining; e, f, g, h, with HE counterstaining; g and h, enlarged views of e and f, respectively).

**Figure 5:**
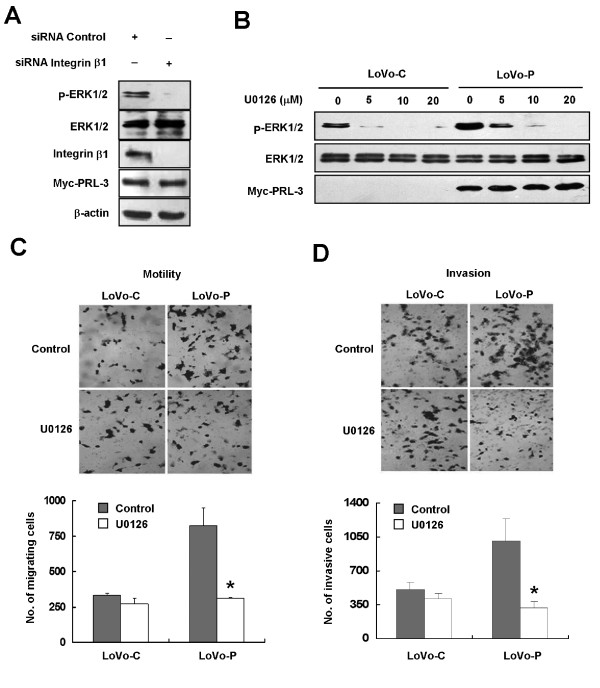
**Integrin β1 mediates PRL-3-induced ERK1/2 activation**. (A) Knockdown of integrin β1 abolished PRL-3-induced ERK1/2 phosporylation. LoVo-P cells were treated with siRNA against integrin β1 or control for 72 h, and then lysates (50 μg of protein) were subjected to Western blot to analyze the expression of p-ERK1/2, ERK1/2, Myc-tag (for Myc-PRL-3), integrin β1, and β-actin (B) U0126 inhibited PRL-3-induced ERK1/2 phosporylation in a dose-dependent manner. Twenty-four hours after plating, LoVo-C and LoVo-P cells were treated with indicated concentration of U0126 for 1 h, and their lysates (50 μg of protein) were analyzed for p-ERK1/2, ERK1/2 and Myc-tag (for Myc-PRL-3) by Western blot. (C) and (D) Inhibition of ERK1/2 activity by U0126 abolished PRL-3-induced cell motility and invasion. LoVo-C and LoVo-P cells were treated with 10 μM of U0126 for 1 h, and then subjected to motility and invasion assays as described in Figure 2B and 2C, respectively. Values were the total number of stained cells. The experiments were repeated at least three times independently. Error bars represent standard errors of the mean value (*, P < 0.05).

We previously found that overexpression of PRL-3 activated ERK1/2 in HEK293 cells [24]. We also obtained a similar result in LoVo cells (Figure 4A). ERK is an important signal transducer triggered by integrin and is closely implicated in ECM-dependent cell motility [33-35]. To examine the relevance of PRL-3 with ERK1/2 activity in colon cancer, expression of PRL-3 and p-ERK1/2 in 11 pairs of consecutive 4-μm primary lesions from sporadic colon cancer patients was evaluated by an immunohistochemical assay with anti-PRL-3 and anti-p-ERK1/2 antibodies, respectively. The representative staining of PRL-3 and p-ERK1/2 in tumor tissue slices is shown in Figure 4B. We found 4 of 5 (90%) PRL-3-expressing samples simultaneously expressed p-ERK1/2, whereas 5 of 6 (83.3%) PRL-3-negative samples were p-ERK1/2-negative either. Statistic analysis (Chi-square test) showed that expression of p-ERK1/2 was positively correlated with that of PRL-3 in colon cancer (see Additional file 3; *, P < 0.05). Interestingly, knockdown of integrin β1 abolished PRL-3-induced phosphorylation of ERK1/2 in LoVo-P cells (Figure 5A), raising the possibility that integrin β1 is an intermediate transducer between PRL-3 and ERK1/2 signaling pathway. Now that depletion of integrin β1 decreased PRL-3-promoted cell motility and invasion and ERK1/2 activation (Figure 2B and 2C) but had no effect on protein level of PRL-3 (Figure 5A), we sought to examine effects of selective inhibition of ERK1/2 in the same cellular context. LoVo cells were treated with 10 μM of the ERK1/2 inhibitor U0126, the concentration of which was sufficient to abrogate ERK1/2 phosphorylation but neither the protein levels of PRL-3 (Figure 5B) nor integrin β1 (data not shown) in LoVo-P cells. We found that ERK1/2 inhibition significantly reduced PRL-3-promoted motility and invasion of LoVo-P cells (*, P < 0.05, Figure 5C, D), while those of LoVo-C cells were not affected. Thus, we conclude that ERK1/2 signaling is also crucial for functions of PRL-3, and importantly, integrin β1 mediates the signaling between PRL-3 and ERK1/2.

### PRL-3 promotes cell invasion by altering the balance between MMP2 and TIMP2

**Figure 6:**
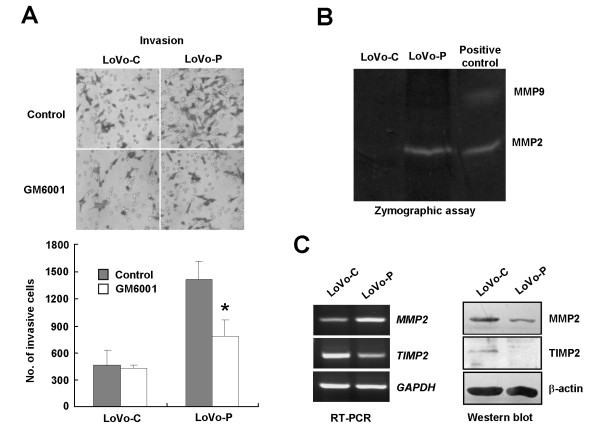
**PRL-3 promotes invasion by enhancing MMP2 activity**. (A) MMP activity was required for PRL-3-mediated invasion. Cells were treated with 25 μM of GM6001 or DMSO (control) for 2 h, and analyzed for their invasion abilities with Matrigel-coated transwell chambers. The invasion assay was performed for three times independently. Top: Representative illustrations of LoVo cells invading to the underside of filters. Bottom: Total number of invasive cells. The experiments were repeated three times independently. Error bars represent standard errors of the mean value (*, P < 0.05). (B) PRL-3 increased gelatin hydrolytic activity of MMP2 in LoVo cells. Equal amount of conditioned serum-free media of LoVo-C and LoVo-P cells was subjected to a zymographic assay as described in Experimental Procedures. A small aliquot of calf serum containing MMP9 and MMP2 was included as the positive control. Bright bands contrasting to dark background represented the hydrolysis area of gelatin catalyzed by the MMP at the same molecular weight. (C) PRL-3 affected MMP2 and TIMP2 expression at both mRNA and protein levels. Equal amount of cell lysates (50 μg of protein) and RNA samples were subjected to Western blot and RT-PCR assays to analyze the expression of MMP2 and TIMP2, respectively.

We have shown that PRL-3 promoted invasion of LoVo cells through integrin β1-mediated ERK1/2 signaling. Invasion is a key process of cancer cell metastasis. It is involved with secreting proteolytic enzymes, including Matrix metalloproteinase (MMPs), to degrade ECM and basement membranes. MMPs are capable of degrading all components of ECM and play important roles in tumor metastasis [36,37]. It was reported that MMPs could be activated by integrin and ERK signaling [38-41]. Therefore, we first examined the requirement of MMPs activity for PRL-3-mediated invasion. LoVo-C and LoVo-P cells were treated with the MMPs inhibitor GM6001 and analyzed for invasion abilities with transwell chambers. Figure 6A showed that GM6001 significantly reduced PRL-3-induced invasion in LoVo-P (*, P < 0.05), but not in LoVo-C, supporting a functional link between PRL-3 and MMPs activity.

To dissect the mechanism of MMPs in PRL-3-promoted cell invasion, gelatinolytic activities of MMP2 and MMP9, two key members of MMP family, were examined by a zymographic assay. In brief, concentrated and normalized serum-free medium of LoVo-C and LoVo-P cells, which contained MMPs secreted by cells, were subjected to electrophoresis in a 10% gel containing the substrate of gelatin and carried out enzymatic reaction. Bright bands contrasting to dark background indicated the absence of gelatin, which had been hydrolyzed by MMPs running to the corresponding molecular weight. Figure 6B showed that MMP2 activity was strongly increased in the culture medium of LoVo-P cells compared to that of LoVo-C cells. No activity of MMP9 was detected in these cells. It is known that activity of MMP2 is regulated by a dynamic balance between MMP2 and its endogenous tissue inhibitor TIMP2 post-translationally [42,43]. To clarify whether increased MMP2 activity resulted from up-regulation of MMP2 or down-regulation of TIMP2, we examined expression of MMP2 and TIMP2 at both mRNA and protein levels in LoVo-C and LoVo-P cells. MMP2 mRNA was increased in LoVo-P cells, contrary to the decrease of TIMP2 mRNA in LoVo-P cells (Figure 6C, left). At protein level, MMP2 was decreased and TIMP2 were hardly detected in LoVo-P cells (Figure 6C, right). We reasoned that the decrease of MMP2 protein level of LoVo-P cell lysates might result from enhanced secretion of activated MMP2 into the outside of cells or increase of activation-induced MMP2 proteolysis. Therefore, PRL-3 might alter the balance between MMP2 and TIMP2 to facilitate MMP2 activation at multiple levels.

## Discussion

Protein kinases and phosphatases regulate multiple physiological processes [44,45]. Phosphatases usually function as tumor suppressors, but some of them have stimulatory effects on cancer-associated processes[46]. PRL-3 is a metastasis-promoting phosphatase [47,48]. It has been found to promote metastasis of a variety of cells, including Chinese hamster ovary cell CHO, mouse melanoma cell B16, and gastric cancer cell SGC7901 [18,19,49].

In this study, we examined the roles of integrin β1-ERK1/2 signaling in PRL-3-facilitating metastasis using human colon cancer cell LoVo, colon cancer tissues from patients, and a metastatic mouse model. We found endogenous integrin β1 was associated and colocalized with exogenous PRL-3 in LoVo cells. We tried to explore whether there is a direct interaction between these two molecules by an in vitro binding assay with purified recombinant PRL-3 and cytoplasmic domain of integrin β1, however, no interaction was found (data not shown). It's possible that integrin α1 mediated PRL-3-integrin β1 interaction, because we previously showed that PRL-3 physically interacted with integrin α1 in HEK293 cells [24]. Unfortunately, integrin α1 protein was not detected in LoVo cells. Whereas in both LoVo-P cells and gastric cancer cells BGC823 stably expressing PRL-3 (BGC823-P), which have detectable integrin α1 on the cell membrane, we observed PRL-3-integrin β1 interaction (data not shown), suggesting that such interaction might be indirect and integrin α1-independent, at least for these two cell lines. Besides α1, integrin α2-9 and α V are also integrin β1-binding proteins [33]. Their roles in mediating the PRL-3-integrin β1 interaction deserve further exploration.

Here we demonstrated that stable expression of PRL-3 decreased tyrosine phosphorylation of integrin β1. Tyrosine phosphorylation of integrin β1 has been reported to impair its binding ability with talin [50]. Another study showed that tyrosine dephosphorylation of integrin β1 altered its association with actin [51]. Recently, a large-scale survey of tyrosine kinase activity in non-small cell lung cancer cell lines identified Y783 of integrin β1 as a potential phosphorylation site [52]. However, kinases and phosphatase responsible for tyrosine phoshorylation modification of integrin β1 are unknown. Therefore, it remains to be determined whether phosphorylation modification of integrin β1 is critical for its signaling transduction and necessary for functions of PRL-3 or whether integrin β1 is a substrate of PRL-3.

We also revealed a PRL-3-integrin β1-ERK1/2 pathway in controlling motility and invasion of colon cancer cell LoVo. We showed that both activation of ERK1/2 and the presence of integrin β1 were necessary for PRL-3 to promote motility and invasion. Activation of ERK1/2 by PRL-3 is dependent on integrin β1. Moreover, knockdown of integrin β1 efficiently inhibited PRL-3-mediated lung metastasis of LoVo cells in nude mice with a comparable effect to that of silencing of PRL-3. However, the intermediate signaling events between integrin β1 and ERK1/2 are still unclear. Activation of ERK is stimulated by both soluble growth factors and integrin-mediated adhesion signals. Integrins intersecting the ERK/MAPK pathway at multiple level, and the crosstalk between growth factors and integrin signaling, give rise to complicated integrin signaling networks and distinctive ERK activation signals [53-55]. To find the intersection point of PRL-3 in the integrin signaling networks would contribute to clarifying the PRL-3 promoted motility, invasion and metastasis. Interestingly, PRL-3 has been reported to activate Src kinase to initiate signaling events, culminating in pathways of ERK1/2, Stat3, and p130cas [21]. Src is one of downstream factors of integrin β1 signaling as well as a upstream molecule of ERK activation [56]. Therefore, whether PRL-3 activates ERK1/2 through the integrin β1-Src pathway or others, such as the integrin β1-Grb2 pathway, deserves further exploration. Downstream of the PRL-3-integrin β1-ERK1/2 pathway, we found that MMP2 exerted proteolysis function on ECM, a critical event for cancer metastasis. PRL-3 enhanced gelatin hydrolytic activity of MMP2 by increasing MMP2 mRNA and decreasing TIMP2 mRNA and protein. The imbalance of MMP2/TIMP2 expression might account for high MMP2 enzymatic activity imposed by PRL-3 overexpression. In a recent study, PRL-3 expression level was found to be positively correlated with MMP2 activity in high grade of glioma tissues [57], supporting our findings about MMP2 activation in LoVo cells. The precise mechanism of PRL-3 in regulating MMP2 remains to be further clarified.

## Conclusion

Taken together, our results suggest that PRL-3's roles in motility, invasion, and metastasis in colon cancer are critically controlled by the integrin β1-ERK1/2-MMP2 signalings. Deeper dissecting the regulation of the PRL-3-integrin β1-ERK1/2-MMP2 pathway may have a therapeutic implication for prognosis and treatment for colon cancer metastasis.

## Abbreviations

ERK: extracellular signal-regulated kinase; ECM: extracellular matrix; FAK: focal adhesion kinase; GFP: green fluorescent protein; Grb2: Growth factor receptor-bound protein 2; MAPK: mitogen-activated protein kinases; MMP: matrix metalloproteinase; PRL-3: phosphatase of regenerating liver-3; RT-PCR: reverse transcription-polymerase chain reaction; shRNA: short hair-pin RNA; siRNA: small interfering RNA; Src kinase: sarcoma kinase.

## Competing interests

The authors declare that they have no competing interests.

## Authors' contributions

LP participated in experimental design, carried out molecular and biochemical analyses, performed in vitro invasion, motility, and animal studies and statistical analysis, interpreted the data and wrote the manuscript. XX participated in molecular and biochemical analyses, performed in vitro invasion, motility, wound healing, and immuno-histochemical studies and the statistical analysis. WL participated in molecular and biochemical analysis. LQ carried out experimental design and coordination, interpreted the data and wrote the manuscript. LM participated in molecular and biochemical analyses, participated in experimental coordination. SL, BJ and JW participated in molecular and biochemical analyses. CS conceived of the study, carried out experimental design, interpreted the data and wrotet the manuscript. All authors read and approved the final manuscript.

## Supplementary Material

Additional file 1 **Western blot analysis of PRL-3 protein in LoVo cells**. LoVo-C and LoVo-P cells were treated with 2 μm Adriamycin or DMSO for 12 h, then cells were harvested and cell lysates (50 μg of protein) were subjected to Western blot with antibodies against PRL-3 (Sigma), Myc, p53 (DO-1, Santa Cruz), and GAPDH, respectively. Antibody to PRL-3 recognized exogenous PRL-3, but no endogenous PRL-3 was detected in both cell lines. LoVo-P cells had more p53 expression, which was consistent with results from Basak et al [Ref [23]]. Click here for file

Additional file 2 **Wound healing assay**. The effect of PRL-3 on the motilities of LoVo cells analyzed with wound healing assay for 72 h and the closure of the wound line was recorded every 24 h. Click here for file

Additional file 3 **Correlation between Expression of PRL-3 and P-ERK1/2 in Human Colon Cancer Tissues**. File shows the correlation between expression of PRL-3 and P-ERK1/2 in human colon cancer tissues. Click here for file
