# Supplementary material for: PRL-3 promotes the motility, invasion, and metastasis of LoVo colon cancer cells through PRL-3-integrin β1-ERK1/2 and-MMP2 signaling
Source: Mol Cancer. 2009 Nov 24;8:110. doi: 10.1186/1476-4598-8-110 (PMC2792223; doi:10.1186/1476-4598-8-110)
Supplement: Additional file 3 — Correlation between Expression of PRL-3 and P-ERK1/2 in Human Colon Cancer Tissues. File shows the correlation between expression of PRL-3 and P-ERK1/2 in human colon cancer tissues. [file 1476-4598-8-110-S3.DOC]

**Table S1.** Correlation between Expression of PRL-3 and P-ERK1/2 in 11 Human Colon Cancer Tissues

| P-ERK1/2 | PRL-3 | Total | Correlation |
| --- | --- | --- | --- |
|  | Positive Negative |  | *R P* value |
| Positive | 5 1 | 6 | .633 .036 |
| Negative | 1 4 | 5 |  |
| Total | 6 5 | 11 |  |
| Statistical analysis was performed by the Chi-square test | | | |
